# Immunotherapy resistance in esophageal cancer: Possible mechanisms and clinical implications

**DOI:** 10.3389/fimmu.2022.975986

**Published:** 2022-09-02

**Authors:** Pinhao Fang, Jianfeng Zhou, Zhiwen Liang, Yushang Yang, Siyuan Luan, Xin Xiao, Xiaokun Li, Hanlu Zhang, Qixin Shang, Xiaoxi Zeng, Yong Yuan

**Affiliations:** ^1^ Department of Thoracic Surgery and Institute of Thoracic Oncology, West China Hospital, Sichuan University, Chengdu, China; ^2^ West China Biomedical Big Data Center, West China Hospital, Sichuan University, Chengdu, China

**Keywords:** immunotherapy, esophageal cancer, intrinsic resistance, acquired resistance, biomarker

## Abstract

Esophageal cancer (EC) is a common malignant gastrointestinal (GI) cancer in adults. Although surgical technology combined with neoadjuvant chemoradiotherapy has advanced rapidly, patients with EC are often diagnosed at an advanced stage and the five-year survival rate remains unsatisfactory. The poor prognosis and high mortality in patients with EC indicate that effective and validated therapy is of great necessity. Recently, immunotherapy has been successfully used in the clinic as a novel therapy for treating solid tumors, bringing new hope to cancer patients. Several immunotherapies, such as immune checkpoint inhibitors (ICIs), chimeric antigen receptor T-cell therapy, and tumor vaccines, have achieved significant breakthroughs in EC treatment. However, the overall response rate (ORR) of immunotherapy in patients with EC is lower than 30%, and most patients initially treated with immunotherapy are likely to develop acquired resistance (AR) over time. Immunosuppression greatly weakens the durability and efficiency of immunotherapy. Because of the heterogeneity within the immune microenvironment and the highly disparate oncological characteristics in different EC individuals, the exact mechanism of immunotherapy resistance in EC remains elusive. In this review, we provide an overview of immunotherapy resistance in EC, mainly focusing on current immunotherapies and potential molecular mechanisms underlying immunosuppression and drug resistance in immunotherapy. Additionally, we discuss prospective biomarkers and novel methods for enhancing the effect of immunotherapy to provide a clear insight into EC immunotherapy.

## Introduction

According to a new global report (1), Esophageal cancer (EC) is the ninth most common malignant tumor and the sixth most common cause of cancer-related deaths. The two main pathological subtypes of EC are esophageal squamous cell carcinoma (ESCC) and esophageal adenocarcinoma (EAC). Unfortunately, because the early symptoms of EC are easily neglected and because the biological characteristics of EC are invasive, patients are often diagnosed at a late stage, with only a 30% five-year survival rate (2). Surgery combined with neoadjuvant chemoradiotherapy (nCRT) remains the first choice of treatment for patients with locally advanced-stage EC. Despite advances in nCRT and surgical therapy, many patients continue to progress to tumor metastases and recurrence. Moreover, side effects limit the use of chemoradiotherapy. Novel therapies against EC are necessary to improve the prognosis of patients with EC (3).

Immunotherapy is a series of treatments aimed at enhancing the strength of the immune system to act against cancer cells by modifying signaling pathways (4). To date, immune checkpoint inhibitors (ICIs) have been applied to treat cancer; they target the suppressed immune system to activate the tumor-cell-killing capacity of immune cells (5). ICIs and autologous T cells expressing chimeric antigen receptors (CAR), the most commonly used immunotherapies, have been evaluated in various cancers (6, 7). In recent decades, immunotherapy has become a prospective option for patients with EC, and increasing evidence has shown that immunotherapy has been successfully used in treating solid and hematologic malignancies and improving patient management. However, immunotherapy resistance has become an extreme challenge that impairs the effects of immunotherapy. Although success has been achieved in the field of immunotherapy for treating patients with EC, most patients do not respond well to immunotherapy, mainly because of both intrinsic and acquired immune resistance. Intrinsic immunotherapy resistance involves innate elements, including normal immune cells and molecules that exhibit mutual interaction during immune progression and inhibit the anti-tumor response. Besides the co-affection of immune cells and molecules, the characteristics of tumor cells also play an important role in intrinsic immunotherapy resistance, although the exact mechanism is still unclear (8). According to recent studies, patients with tumors who were initially responsive to immunotherapy were prone to developing acquired resistance (AR). In particular, in gastrointestinal (GI) cancer, the rate of AR is above 50%. Therefore, clinical researchers need to investigate the potential mechanism of immune resistance in EC and identify novel immunotherapy resistance biomarkers. In this review, we summarize advances in immunotherapy for patients with EC, including ICIs, CAR-T cell treatment, and tumor vaccines that stimulate the immune system and anti-tumor response. In addition, we discuss the mechanism of immunosuppression and drug resistance in EC, prospective biomarkers for predicting immunotherapy resistance, and novel clinical strategies for overcoming immunotherapy resistance.

## Immunotherapies for EC

### Immune checkpoint inhibitors

**Figure 1:**
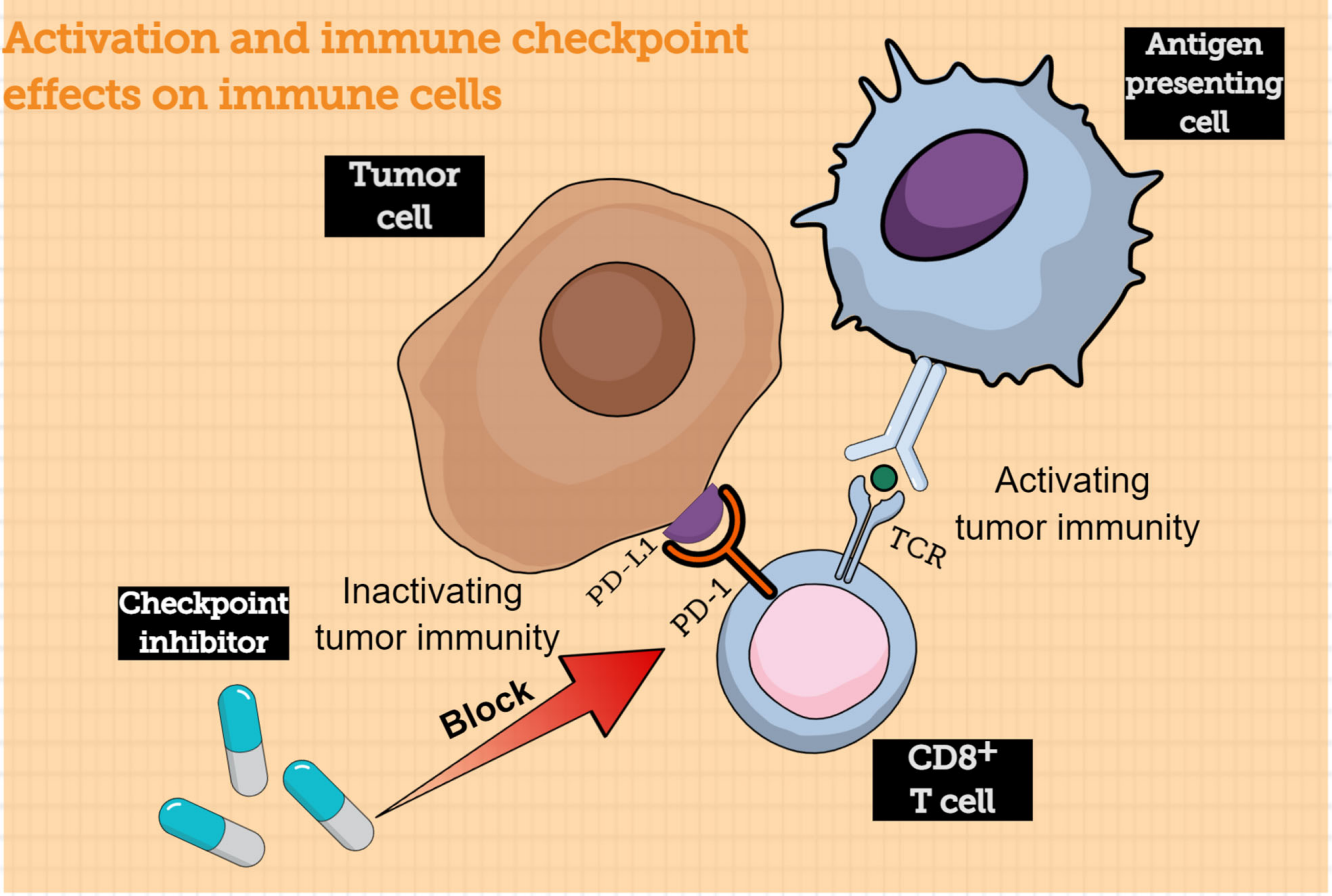
Activation of immune-checkpoint effects on immune cells. The CD8+ T cells can be activated by interacting with antigen-presenting cells, following which the CD8+ T cells can acquire the capacity to kill tumor cells. However, tumor cells express immune checkpoint proteins that bind to receptors on the surface of CD8+ T cells to evade immune cells; immune checkpoint inhibitor can block this process, thereby allowing CD8+ T cells to kill tumor cells. TCR, T cell receptor; PD-1, programmed cell death protein 1; PD-L1, programmed cell death ligand 1.

Traditionally, antigen-presenting cells (APCs) could submit the major histocompatibility complex (MHC) to T cells. When the T cell receptors (TCR) bind with the submitted MHC, CD8+ T cells are activated and converted into tumor cell killers. Inversely, to protect our system from being harmed by an “overprotective” immune response, the immune checkpoints play a vital role in immunosuppression and act as “inhibitors” to prevent long-lasting inflammation and autoimmunity (9). Programmed cell death protein 1 and programmed cell death ligand 1 (PD-1/PD-L1) are common immune checkpoints in T-cell activation. PD-1 is often expressed on the surface of various immune cells, such as T cells; when it binds to its ligand PD-L1, which is often abnormally highly expressed on tumor cells, the intercellular inhibition signaling pathways of T cells are activated, and the T-cell effect is suppressed (10). Moreover, the PD-1/PD-L1 axis could mediate the process of immune monitoring and play a vital role in tumor progression (11). PD-L1 expression by tumor cells could protect them from lysis mediated by CD8+ T cells (12). When engaged by PD-L1, activated T cells could express CD80, which acts as a receptor delivering a suppression signal, leading to peripheral T-cell tolerance (13). During prolonged exposure to a tumor antigen, T cells upregulate negative regulators such as PD-1, leading to their functional exhaustion (14). Antibody-based immunotherapy that blocks this signaling pathway is a prospective treatment for tumors (**Figure 1**). Anti-PD-1 antibody development has become a hot spot in the immunotherapy field; this strategy has been proven effective in melanoma, non-small-cell cancer, and renal-cell cancer, exhibiting ideal objective response rates. The combined positive score (CPS) is often used by clinicians to evaluate the expression of PD-L1; the value of CPS is calculated using an immunohistochemical scoring algorithm.


CPS=(total number of PD−L1−stained cells/total number of tumor cells)× 100,

where the maximum score is 100 and CPS >1 is considered positive in EC (15). On the basis of the CPS results, patients with PD-L1-negative tumors have been shown to be more likely to exhibit a non-objective response compared with those with PD-L1-positive tumors (16). Recently, clinical trials have evaluated PD-1/PD-L1 inhibitors for EC treatment and have achieved favorable therapeutic effects. Pembrolizumab, a classic high-affinity monoclonal PD-1 antibody, has been shown to result in survival benefits in patients with various tumors. A global multicenter, randomized-control phase III clinical trial enrolled 628 patients with advanced EC who received pembrolizumab therapy; the final trial results showed that patients with PD-L1 CPS ≥10 may benefit more from pembrolizumab than from chemotherapy and that pembrolizumab could prolong the overall survival (OS) of patients with advanced EC (17). Meanwhile, the KEYNOTE-590 clinical trial proved that pembrolizumab combined with chemotherapy could provide superior OS, progression-free survival (PFS), and overall response rate (ORR) compared to chemotherapy alone in patients with advanced EC (18). Pembrolizumab combined with chemotherapy may likely achieve better survival outcomes and may become a new standard treatment for patients with advanced EC. Pembrolizumab, utilized as a second-line therapy for EC in different clinical trials, has shown positive clinical effects in both patients with ESCC and those with EAC (19). A phase II clinical trial included 30 eligible patients with locally advanced or metastatic EC who received camrelizumab (SHR-1210, anti-PD-1) and apatinib (anti-angiogenesis) in combination with chemotherapy. The study results demonstrated the feasible anti-tumor activity of immune checkpoint inhibitors combined with anti-angiogenesis treatment and chemotherapy (20). In the clinical trial ATTRACTION-3, nivolumab, another immune checkpoint inhibitor, was proven to produce an improvement in OS and exhibit better safety compared with traditional chemotherapy in patients with advanced ESCC (21). These extensive clinical trials have demonstrated the superiority of PD-1/PD-L1 checkpoint inhibitors, which exhibit better effectiveness and fewer side effects than conventional chemotherapy. PD-1/PD-L1 checkpoint inhibitors may become a novel prospective therapeutic option for patients with EC. Meanwhile, clinical trials are well underway for various novel PD-1 antibodies, including JS001, durvalumab, and other novel immune drugs against EC, and the results of their therapeutic effects are expected (22–25).

In addition to PD-1/PD-L1, another well-recognized immune checkpoint is T lymphocyte-associated antigen 4 (CTLA4), which is commonly expressed on regulatory T cells (Tregs) and activated T lymphocyte surfaces; it acts as a vital element in T-cell self-tolerance and regulation. Many studies have verified that the overexpression of CTLA4 is associated with T-cell cycle arrest, reduced interleukin-2 (IL-2) expression, and arrested T cell G1 phase (26). Consequently, the function of T cells is reduced, causing the immune evasion of cancer cells. Remarkably, this key immune checkpoint has been used as a therapeutic target in the domain of anti-tumor drugs and immunotherapy (27). A previous study showed that CTLA4 is expressed not only by T cells but also by tumor cells, which indicates that the exact function of CTLA4 is unknown (28). The main representative drugs for CTLA4 target therapy in the clinic are ipilimumab and tremelimumab. CTLA4 checkpoint inhibitor treatment in patients with EC could provide favorable survival benefits and reduce treatment-related adverse events. A phase II clinical trial investigated the CTLA4 inhibitor tremelimumab for patients with gastric cancer (GC) and EAC; a small cohort of patients received a significantly long-lasting benefit and acquired clinical benefit with mild drug-related toxicity. However, the response rate to tremelimumab was only 5% (29). Owing to the limited number of clinical trials investigating CTLA4 inhibitors in EC, detailed information on the efficiency, safety, and side effects of tremelimumab still need to be determined.

### Chimeric antigen receptor T-cell therapy

Tumor cells are highly immunogenic, with the specific expression of tumor-associated antigens (TAA), which are pivotal in activating anti-tumor immune responses. T cells can recognize tumor cells based on TAA molecules and attack tumor cells. Chimeric antigen receptor T-cell (CAR-T-cell) therapy is a type of immunotherapy based on this mechanism. CAR-T-cell therapy refers to the genetic engineering of T-cell antigen receptors. During the process of modification, patient cells are first isolated from peripheral blood and engineered *ex vivo* to generate chimeric receptors that specifically recognize TAAs. Therefore, CAR-T cells possess tumor-recognizing characteristics, and they can be infused back into the blood of patients as an anti-tumor therapy (30). CAR-T cells typically consist of four fragments. The extracellular domain is a variable segment that originates from an antibody that acts as a TAA recognizer. A spacer modulates the distance between tumor and CAR-T cells and connects them to the transmembrane domain. The transmembrane domain can deliver the signal to the intercellular signaling domain, which is mainly composed of CD3ζ, and then activate T cells when engaged with tumor cells through TAAs expressed on the surface of tumor cells (31, 32). CAR-T immune therapy is commonly used in hematologic malignancies and has been proven to be effective in patients with diffuse large B-cell lymphoma (33) and leukemia (34). In the past few years, CAR-T immune therapy has also been explored as a treatment for solid tumors, including EC. According to previous studies, the overexpression of erythropoietin-producing hepatocellular receptor A2 (EphA2) could facilitate carcinogenic effects in various tumors (35); furthermore, EphA2 overexpression has been detected, which is associated with poor prognosis in ESCC (36). Shi et al. constructed EphA2-targeting CAR-T cells that showed a better ability to kill ESCC cells and promote cytokines *in vitro* (37). Another well-known TAA is the human epidermal growth factor receptor 2 (HER2), which is highly expressed in both breast cancer and EC. In an *in vitro* experiment, Yu et al. successfully developed CAR-T cells targeting the HER2 antigen. CAR-T cells showed a strong anti-tumor effect *in vitro*, significantly suppressed tumor growth in xenograft mice, and demonstrated the ability to specifically kill HER2-positive EC cells (38). Additionally, studies have shown that engineered CAR-T cells targeting mucin 1 (MUC1) and CD276 can induce the release of high levels of cytokines, achieving better persistence and durability to regulate a stronger anti-tumor response in a subcutaneous xenograft mouse model of EC (39, 40); this indicates that CAR-T cell therapy merits testing in EC clinical trials in the future. Various preclinical studies have identified novel methods for enhancing the anti-tumor effect of CAR-T cells. Recently, a new generation of CAR-T cells was designed by encoding a truncated cytoplasmic domain that binds to CD3z and CD28 domains together; the modified CAR-T cells showed better persistence and anti-tumor effects than traditional CAR-T cells (41). Zhang et al. designed enhanced CAR-T cells targeting MUC1, which is a complex glycoprotein overexpressed in EC that additionally activates the JAK-STAT signaling pathway. The strengthened MUC1-CAR-T cells survived longer in mice and appeared to exhibit a high treatment efficiency (39). Although many preclinical experiments have proved that CAR-T cells are a prospective therapeutic candidate against EC, no CAR-T-cell therapy has been applied in clinical trials for patients with EC. Additional breakthroughs are of great necessity in the clinical translation of CAR-T-cell therapy.

### Tumor vaccines

As described previously, high immunogenicity of TAAs has been identified in EC. Several TAAs are highly expressed in EC, among which the most common TAAs have been confirmed in EC till date, including New York esophageal squamous cell carcinoma 1 (NY-ESO-1), TTK protein kinase (TTK), cancer-testis antigen 2 (CTAG2), and melanoma-associated antigen-A (MAGE-A) (42). Furthermore, the anti-tumor effects or immune-cell reactions to these TAAs could be tested in EC samples from patients. Chen et al. proved that MAGE-A3-specific CD8+ T cells could be isolated from the peripheral blood of patients with EC and that CD8+T cells could react with MAGE-A3 peptide; consequently, these CD8+T cells could specifically lyse certain tumor cells (43). Another study confirmed that the NY-ESO-1 dominant B-cell epitope and NY-ESO-1 antibody could be detected in the serum of patients with various cancers (44). Cancer vaccines, based on immune reactions through specific TAAs, have become a hot topic in cancer therapy; they act by stimulating T cells to exert anti-tumor effects and kill tumor cells. Several peptide vaccines have been tested in clinical trials. Sipuleucel-T, a cancer vaccine, has been shown to exhibit therapeutic effects in prostate cancer by prolonging the overall survival of patients with prostate cancer (45). Additionally, peptide vaccines in patients have shown a good therapeutic effect. Kageyama et al. conducted a clinical trial enrolling 25 patients with advanced EC subcutaneously injected with a cholesteryl pullulan-NY-ESO-1 (CHP-NY-ESO-1) complex vaccine, and no adverse events were observed during the treatment period. The vaccine can induce specific immune responses and provide a better survival benefit in patients with advanced EC (46). Chemoradiation therapy in combination with multiple peptide vaccines (kinase of the outer chloroplast membrane 1 (KOC1)), upregulated lung cancer 10 (URLC10, TTK, VEGFR1, and VEGFR2) showed a superior effect and a satisfactory level of safety in patients with unresectable ESCC (47). However, tumor vaccines have not been commonly utilized in EC clinical practice thus far, and the mechanism underlying their anti-tumor effect needs further study.

The current advancements in immunotherapy for EC are summarized in **Table 1**. Immunotherapy has been successfully used in clinics, especially in the field of GI cancer, and it has become a prospective approach against malignancies. Immunotherapy has achieved a significant breakthrough in treating EC, gastric cancer, and colorectal cancer during the past decade, which has brought new hope to cancer patients. Unfortunately, the overall response rate (ORR) of immunotherapy is lower than 30%, and patients who initially respond to immunotherapy are likely to progress to AR (49–51). Moreover, approximately 70% patients appear to exhibit primary resistance to immunotherapy or even develop a hyper-progressive disease, the durability and effect of immunotherapy are extremely reduced. Therefore, clarifying the potential molecular mechanisms involved in immunosuppression is important for selecting preferable strategies for EC immunotherapy.

**Table 1:** Current advancements in immunotherapy for EC.

Target	Mechanism	Drug or Treatment	Study type	Reference
PD-L1	Expressed on the surface of EC cells, when binding with PD-1, the activation of T cells is inhibited and cause immune escape	Pembrolizumab	Clinical research	(17–19)
PD-1	The receptor of PD-L1 expressed on the surface of T cells, negatively regulates T cells	Camrelizumab	Clinical research	(20)
		Nivolumab	Clinical research	(21, 48)
		Durvalumab	Clinical research	(24)
		JS001	Clinical research	(25)
CTLA4	Associated with T cell cycle blocked which can lead the T cells G1 phase arrested	Tremelimumab	Clinical research	(29)
		Ipilimumab	Clinical research	(48)
EphA2	Related to poor degree of tumor differentiation and lymph node metastasis in EC	EphA2 targeting CAR-T cells	Basic experiment	(37)
HER2	Highly expressed in EC and associated with poor prognosis	HER2 targeting CAR-T cells	Basic experiment	(38)
MUC1	High expression of MUC1 was associated with tumor size, lymph node metastasis and distant metastasis in EC	MUC1 targeting CAR-T cells	Basic experiment	(39)
CD276	Promotes glucose metabolism in tumor and inhibits the function of CD8+ T cells	CD276 targeting CAR-T cells	Basic experiment	(40)
NY-ESO-1	One of TAAs expressed by EC cells	Tumor vaccines	Clinical research	(46)
KOC1	One of TAAs expressed by EC cells	Tumor vaccines	Clinical research	(47)
TTK	One of TAAs expressed by EC cells	Tumor vaccines	Clinical research	(47)

PD-L1, programmed cell death ligand 1; PD-1, programmed cell death protein 1; CTLA-4, cytotoxic T lymphocyte-associated protein 4; EphA2, hepatocellular receptor A2; HER2, human epidermal growth factor receptor 2; MUC1, mucin 1; NY-ESO-1, New York esophageal squamous cell carcinoma 1; KOC1, kinase of the outer chloroplast membrane 1; TTK, TTK protein kinase; EC, esophageal cancer; CAR-T, chimeric antigen receptor T cell; TAA, tumor-associated antigen.

## Potential mechanisms of resistance to immunotherapy in EC

EC cells can abnormally express specific antigens, which can be recognized by immune cells to initiate an anti-tumor immune response. Traditionally, the response of CTLs activated by APCs has been key for eliminating tumor cells. Dendritic cells (DCs), another participant in the immune response, play a vital role in tumor cell antigen delivery, presenting tumor antigens and rendering CTLs capable of killing tumor cells (52). However, EC cells have undergone mutations to evade human immune cells and resist attack by the immune system.

### Intrinsic resistance

Several factors are involved in immune resistance in EC. A main strategy used by EC cells to escape the immune response is to upregulate immune checkpoint molecules and downregulate tumor antigens. Immune checkpoints, including PD-1, PD-2, and CTLA-4, are usually expressed on the surface of immune cells. These molecules act as critical molecules to prevent immune cells from inducing inflammation, destruction, and autoimmunity. They can block signaling within T cells when triggered. However, tumor cells may highly express these checkpoint proteins to protect themselves from being lysed by CTLs and escape death (53). To date, studies have verified that many checkpoint inhibitory molecules are upregulated by EC cells. The well-studied inhibitory receptors PD-1 and CTLA-4 are commonly detected in EC (54–56). PD-L1 can even be secreted by tumor cells through exosomes to suppress T-cell immunity, which cannot be restored by ICIs (57). Other inhibitory molecules such as lymphocyte-activation gene 3 (LAG-3) and mucin-domain containing-3 (TIM-3) have been demonstrated to be associated with PD-L1 expression in EAC (56). Recently, indoleamine 2,3-dioxygenase 1 (IDO1), a primary enzyme that produces kynurenine and tryptophan to suppress the immune response, has aroused research interest with respect to EC. Kiyozumi et al. conducted a study involving immunostaining of EC tissues from 305 patients with EC and proved that IDO1 showed an inverse correlation with CD8+ expression, indicating that IDO1 may act as a negative factor in immune regulation (58). In addition to CD8+ T cells, macrophages offer great promise as effectors in the anti-tumor immune response because of their strong ability to perform phagocytosis. CD47 is a critical molecule in the regulation of macrophages, and it acts as an immune checkpoint (59). Early studies described CD47 as a “marker of self,” which is a glycoprotein on the surface of red blood cells that protects normal cells from innate immune cells that attack certain hematologic malignancies and solid tumors (60, 61). When activated, CD47 delivers inhibitory signals through signal regulatory protein alpha (SIRPα), a receptor on the surface of macrophages and myeloid cells, impairing the phagocytic activity of macrophages. Thus, the CD47/SIRPα axis serves as a specific myeloid immune checkpoint (62). However, studies have reported that tumor cells can highly express CD47, and abnormal activation of the CD47/SIRPα axis by tumor cells may inhibit the anti-tumor immune response and upregulate the threshold for macrophage phagocytosis (63). Tao et al. demonstrated that the expression level of CD47 is negatively associated with CD8+ T-cell density in ESCC tissues. Additionally, in a preclinical study, they demonstrated that anti-CD47 therapy enhanced the proinflammatory response of immune cells and then CD8+ T cell infiltration density increased in ESCC tissue *in vivo* (63), indicating that the CD47/SIRPα axis might serve as a novel immunotherapeutic target for patients with ESCC. However, the expression of inhibitor molecules on the cancer cell surface has been shown to present high heterogeneity (64). Additionally, the expression level of immune checkpoints could vary among different pathological subtypes (65). Therefore, identifying a reliable immune therapy that targets a certain immune checkpoint remains a severe challenge.

In addition to inhibitory molecule expression, EC cells may secrete cytokines and growth factors to facilitate tumor growth and reduce the anti-tumor immune response. Transforming growth factor-β (TGF-β), a factor secreted by tumor cells (66), plays an important role in immune tolerance by regulating several types of immune cells (67). It is vital for enhancing immune suppression in the tumor microenvironment (TME). Previous clinical studies in patients with EC showed that the TGF-β signaling pathway was abnormally hyperactivated (68), and the expression level of TGF-β was significantly associated with the prognosis of patients with EC (69). TGF-β can directly activate regulatory Tregs to inhibit the cytotoxicity of effector T cells, natural killer (NK) cells, and the antigen-presenting function of DCs. Furthermore, TGF-β can block the differentiation of naïve T cells into effector T cells. Therefore, TGF-β has a complex negative impact on the immune system (70). Cancer cells produce TGF-β and use it for tumor growth (71). TGF-β can decrease the level of IL-2, a cytokine that elicits CD4+ T-cell proliferation (72). Li et al. demonstrated that TGF-β signaling can also affect B-cell-mediated immune regulation. When exposed to EC-derived microvesicles (Mvcs), naive B cells are likely to differentiate into TGF-β-producing cells, thereby suppressing the proliferation of CD8+ T cells (73). Several studies have suggested that cancer-associated fibroblasts (CAFs), characterized by high levels of α-smooth muscle actin and fibroblast protein-α, play a prominent role in supporting tumor growth. TGF-β may also be involved in crosstalk between EC cells and CAFs. TGF-β is highly expressed in patients treated with conventional chemotherapeutic medicine, indicating that chemotherapy may upregulate the level of TGF-β and inhibit the immune response (69). As a well-known cytokine, the interleukin (IL) family plays a significant role in immune cellular signal transduction. IL-6 is the principal factor involved in infection and injury reactions (74). Upon binding to its receptors, IL-6 triggers the pathway and activates downstream molecules, such as STAT1 and STAT3, which may enhance the capacity of tumor cells to survive in a highly inflammatory environment and impair immunotherapy effects (75). Because of its inflammatory effects, IL-6 affects immune resistance in EC. IL-6 originates in the TME, and it is involved in various phenotypes of EC *via* different pathways (76). Upregulation of IL-6 can be found in both ESCC and EAC (77). Meanwhile, high levels of IL-6 promote epithelial-to-mesenchymal transition (EMT), clonogenicity, and chemoresistance in EC (78). IL-6 can inhibit the maturation of DCs through the STAT3 signaling pathway, attenuating anti-tumor immunity (79). In addition, elevated levels of IL-6 secreted from CAFs promote the migration of ESCC cells, and the expression of IL-6 is associated with immunosuppressive phenotypes (80). Additionally, elevated levels of IL-10 have been detected in the serum of patients with ESCC, and the IL-10 level has been positively associated with Treg density (81). IL-10 derived from Treg cells can act along with IL-35 to promote the exhaustion of CD8+ tumor-infiltrating lymphocytes (TILs), thus reducing anti-tumor immunity (82).

In addition to the extensive inhibitory molecules, there are robust suppressive cells in the TME within the EC, which remains a major hurdle in immunotherapy efficiency. As a crucial component of the TME, immune cells are necessary for regulating the anti-tumor response. As a subtype of T cells marked by IL-10 and the transcription factor FOXP3, Tregs are crucial for maintaining self-tolerance. When Tregs are activated by an immune response, inhibitory cytokines such as IL-1 and IL-6 are released into the peripheral blood. Thus, Tregs participate in the suppression of anti-tumor immunity (83). Tregs can be selectively recruited by certain factors to infiltrate the tumor stroma (84, 85), and the degree of infiltration of Tregs is associated with poor prognosis in EC (86). The chemokine (C–C motif) ligand 22 (CCL22) has been proposed to act as a key factor in the aggregation of Tregs. CCL22 released by tumor cells and tumor-infiltrating macrophages attracts the recruitment of Tregs through the combination of C–C chemokine receptor type 4 (CCR4) (87). Additionally, Tregs may recognize tumor antigens such as NY-ESO-1 and suppress specific effector T cells (88). Elevated levels of CCL4 and CCL20 were detected in ESCC tissue together with a high density of CD8+ T cells and Tregs, respectively, showing that Tregs and CD8+ T cells may be correlated through selective recruitment *via* specific expression of CCL20 and CCL4 (89). Immunity suppression in ESCC has been shown to occur because of the specific recruitment of CCL20 to Tregs. Other studies have demonstrated that CCL20 may also attract T helper 17 (Th17) lymphocytes in EC (90), thereby recruiting DCs to promote the activation of CD8+ T cells and enhance anti-tumor immunity (91, 92). Th17 is another subtype of T cell associated with immunity regulation and is commonly recognized as a vital mediator in anti-tumor responses and inflammation (93). Th17 cells can be found at elevated levels in the tumor tissues and peripheral blood of patients with EC (90). Th17 cells secrete the inflammatory cytokine IL-17 to enhance the invasiveness of EAC cells through the NF-κB pathway (94). However, IL-17 might also play a protective role by augmenting the expression of cytotoxic molecules to strengthen the tumor-killing effects of NK cells and promote DC infiltration to recruit immune cells in ESCC (92). Therefore, CCL20 and Th17 may play a dual role in tumor immunity and provide a deeper understanding of the role of CCL20 and Th17 in the immune response. Quezada et al. showed that CTLA-4 can be stably expressed by Tregs (95). Meanwhile, anti-CTLA-4 therapy decreased the number of Tregs in tumor tissues, and it was significantly associated with favorable clinical events, implying that Tregs may mutually affect immune checkpoint molecules in immune regulation (96).

As another vital element consists of the immune inflammatory cells in the TME. Macrophages impact the immune system and affect tumor progression. The degree of tumor infiltration by tumor-associated macrophages (TAMs) has been verified to correlate with prognostic outcomes in some malignancies (97). In oncology, TAMs are traditionally divided into two subgroups with different functions in tumor progression. One subtype is tumor-suppressive macrophages (M1), and the other is tumor-promoting macrophages (M2), characterized by the expression of CD163 and CD204 (98). M1 macrophages play a role in tumor inhibition, whereas M2 macrophages facilitate tumorigenesis. M2 macrophages are generally believed to act as negative regulators of the anti-tumor response. However, the underlying mechanisms remain largely elusive. A high density of M2-like TAMs was greatly associated with high levels of PD-L1 expression, and M2-like TAMs secrete TGF-β, indicating the protective function of M2-like TAMs in immune rejection (99, 100). Additionally, the c-Jun NH2 kinase (JNK) signaling pathway has been identified as a key factor in the transition of macrophages from anti-tumorigenic to tumorigenic, activating M2-like TAMs to release CCL17 and CCl22 in Treg recruitment (101).

Accumulated myeloid-derived suppressor cells (MDSCs) have been detected and verified as indicators of poor prognosis in most patients with EC (102). MDSCs accumulate in response to inflammatory regulators and can obstruct both adaptive and innate anti-tumor immune responses (103). MDSCs impact the anti-tumor response mainly by inhibiting T-cell-regulated tumor clearance (104) but may also act through activation of Tregs (105) and impair innate immunity through mutual effects with macrophages and NK cells. In the presence of MDSC, macrophages are prone to converse into M2 macrophages, and MDSC can also combine with M2 macrophages to block immune surveillance driven by IL-13 (106). The crosstalk between macrophages and MDSC facilitates MDSC IL-10 release and reduces IL-12 production by macrophages (107). In EC, IL-6, CCL2, and aldehyde dehydrogenase 1 (ALDH1) stimulated MDSCs (108, 109). Animal experiments have shown that tumor-derived factors such as IL-6, CXCL16, IFNγ, TNFα, and IGFBP-3 positively regulate the expression of CD38, and high expression of CD38 can enhance the immunosuppressive and tumor-promoting capacity of MDSCs (110).

### Acquired resistance

Immunotherapy induces an anti-tumor response and has been successfully used as a clinical treatment for EC. However, with broader and more frequent use of immunotherapy, an increasing number of patients with EC have had a prolonged time to response; this phenomenon is called AR. However, the exact mechanism of AR in EC remains unknown. Traditionally, the main potential mechanisms of AR are believed to be the loss of T-cell effects and recognition through the downregulation of tumor antigens, enhancement of escape mutation variants, interferon-γ (IFN-γ) signaling, and neoantigen depletion. Evidence has shown that these mechanisms could lead to AR during ICI therapy (51).

When the T-cell functional anti-tumor phenotype is changed and their cytotoxic activity is suppressed, patients who exhibit a primary response to immunotherapy might easily develop AR and progress into tumor relapse. As anti-tumor T cells specifically recognize tumor cells that express a certain antigen, tumor cells may likely progress into AR by decreasing the expression or inducing mutation of their antigens. Previous studies have suggested that T cells activated by ICI therapy preferentially recognize mutational antigens (111). The progression of T-cell activation is largely dependent on the antigens recognizing the major histocompatibility complexes (MHCs) of APCs (112) and tumor cell antigens submitted through MHC class I are regulated by various genes. Thus, when genetic deletions, epigenetic changes, or mutations are caused, these neoantigens presented by APCs are also downregulated, which might result in AR to ICI therapy. Hulpke et al. reported a crucial gene, beta-2-microglobulin (B2M), involved in stabilizing the MHC class I molecules at the cell surface (113). Previously, researchers identified that the loss-of-function mutation B2M was associated with MHC class I dysfunction, which indicated the potential molecular pathway of tumor cells escaping immunity. Restifo et al. first proved that in patients with metastatic melanomas who were treated with immunotherapy, B2M was lost, suggesting that the loss of B2M might be a possible factor that facilitates cancer cell acquisition of immunotherapy resistance (114). In addition, Gettinger et al. found in lung cancer that homozygous loss of B2M could lead to the downregulation of MHC class I in cancer cells. They additionally conducted an *in vivo* experiment by injecting knock-out B2M lung cancer cells into immunocompetent mice that received anti-PD-1 therapy. The results showed that B2M knockout cells were less sensitive to PD-1 blockade than the control group. They additionally proved that CD8+ T cells showed considerably lower cytotoxicity than B2M knockout tumor cells, indicating that B2M could mediate tumor cell escape from ICI therapy through MHC class I expression (115). Meanwhile, an early study conducted by Sade-Feldman et al. showed that B2M alterations were enriched in cancer patients insensitive to anti-CTLA4 therapy compared to responders (116). In EC, Wang et al. observed that B2M could be highly expressed through mesenchymal stromal cells (MSCs), which are considered pivotal cells in the tumor microenvironment of EC. The results of their study suggest that stroma-derived B2M might also be involved in EC immunotherapy resistance and might be a potential mechanism of ICI drug resistance (117).

Another pivotal strategy for activating the anti-tumor response is the JAK-STAT pathway. When IFN-γ is secreted by effector T cells, and it binds to the heterodimeric IFNGR1/IFNGR2, the receptor-associated kinases Janus kinase 1 (JAK1) and Janus kinase 2 (JAK2) are activated (118). Recent clinical studies have demonstrated that suppressing mutations in JAK1 or JAK2 may contribute to drug resistance during ICI therapy (119). Zaretsky et al. reported that patients with melanoma treated with ICIs presented loss-of-function mutations in JAK1 or JAK2, which led to resistance to PD-1 blockade. Additionally, they treated cell lines established from patients with AR with ICIs and demonstrated that the downregulation of the JAK protein was significantly associated with tumor sensitivity to IFN-γ (120). In patients who did not respond to CTLA4 inhibitor therapy, the function of IFN-γ was greatly suppressed (119). Li et al. found that IL-18 is usually downregulated, and the expression of IL-18 was positively correlated with IFN-γ. They verified *in vitro* that deficiency of IL-18 could suppress the cytotoxicity of NK cells and CD8+ T cells, indicating that the absence of IL-18 is likely to mediate the IFN-γ pathway during tumorigenesis in ESCC and lead to AR in anti-tumor immunity (121). Others have reported that long noncoding RNAs (lncRNAs) SNHG20 could serve as a carcinogen in ESCC and affect the JAK-PD-L1 pathway to promote ESCC cell progression (122). However, till date, clinical research on these key signal mutations associated with ICI drug resistance in EC is lacking, and whether additional pathways apart from IFN-γ or JAK are involved in AR to ICI therapy remains unclear.

Mutations frequently occur during the progression of tumor growth, some of which produce neoantigens and affect the response to ICI therapy (51). Previous research has shown that in early lung cancer, CD8+ T cells can react with tumor cells that highly express PD-1. Meanwhile, patients with enriched neoantigen expression appear more sensitive to ICI therapy and acquire more clinical benefits. These results suggest that neoantigen expression levels influence ICI therapy effects (123). Therefore, the loss of mutations in neoantigens through the downregulation of copy number or epigenetic repression may result in immune evasion and resistance to ICIs (124). When stimulating the lost neoantigen *in vitro*, T-cell expansion was observed, indicating that neoantigens may play a vital role in reducing AR to immunotherapy in cancer patients. Notwithstanding that such a mechanism has not been verified in EC, depletion of neoantigens has been verified in lung cancer, indicating that similar mechanisms may also be among other malignancies such as EC, which deserves further exploration and elucidation.

**Figure 2:**
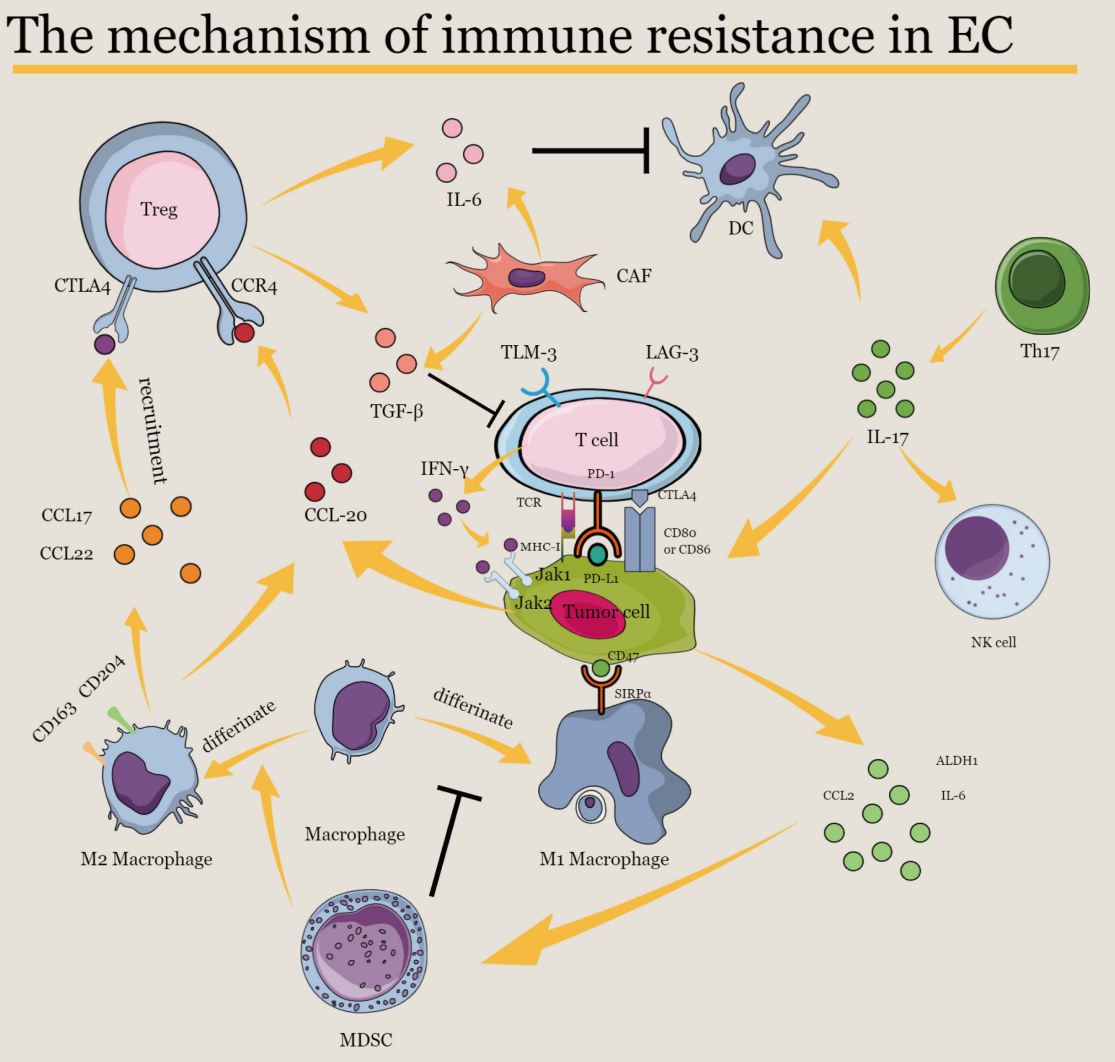
The mechanism of immune resistance in EC. IL-6, interleukin-6; IL-17, interleukin-17; PD-1, programmed cell death protein 1; PD-L1, programmed cell death ligand 1; CTLA-4, cytotoxic T lymphocyte-associated protein 4; CCR4, C–C chemokine receptor type 4; CCL2, C–C motif ligand 2; CCL17, C–C motif ligand 17; CCL20, C–C motif ligand 20; CCL22, C–C motif ligand 22; TGF-β, transforming growth factor-β; TIM-3, T cell immunoglobulin and mucin-domain containing-3; LAG-3, lymphocyte-activation gene 3; IFN-γ, interferon-γ; MHC-I, major histocompatibility complex class I; ALDH-1, aldehyde dehydrogenase 1; SIRPα, signal regulatory protein alpha; JAK1, Janus kinase 1; JAK2, Janus kinase 2; Treg, regulatory T cell; CAF, cancer-associated fibroblast; DC, dendritic cell; NK, natural killer; Th17, T helper 17; MDSC, myeloid-derived suppressor cell.

Although many potential mechanisms involved in primary or acquired resistance to immunotherapy have been discussed above (**Figure 2**), elucidating immunotherapy resistance in EC is extremely challenging because not enough clinical trials apply ICI therapy in EC or to explain the underlying mechanism of immunotherapy and drug resistance. Thus, data from clinical trials and basic experiments are necessary for understanding and overcoming immune resistance and providing more clinical benefits to patients with EC.

## Potential biomarkers of EC immunotherapy

The progression of tumors in patients with EC mainly depends on mutual interactions between tumorigenic EC cells, such as EC cell proliferation and invasion capacities, and the interactivity of immune cells induced by various regulators in the TME. Meanwhile, EC resistance to anti-tumor responses is believed to be a consequence of abnormal production of specific molecules, such as stimulatory and inhibitory factors, or an alteration in the effect of T cells and Tregs. Because of this imbalance in the TME and the high expense of immune therapy, it is particularly necessary to identify reliable biomarkers for predicting the prognosis of patients with EC before treatment with immune therapy. To date, genetic alterations in anti-tumor immunity regulation and TILs have been widely reported.

### Immune checkpoint proteins

PD-L1, also called CD274 or B7 homolog 1, is a transmembrane protein expressed by DCs and EC cells. PD-1 is often expressed on the surface of T cells as a receptor for PD-L1. When it binds to PD-L1, the anti-tumor effect of T cells can be suppressed. The binding of PD-L1 and PD-1 remains the main mechanism of anti-tumor immunity evasion. Immune checkpoint inhibitors can inhibit their binding and help T cells recognize and kill EC cells. According to previous research, the expression of PD-L1 in ESCC ranges from approximately 40% to 80% (125). Most researchers have suggested that the expression level of PD-L1 in EC cells is a reasonable biomarker for predicting the efficiency of PD-L1/PD-1 inhibitors (16, 126). However, the significance of PD-L1/PD-1 expression in both EC tissues and TILs remains controversial. Hatogia et al. reported that high levels of PD-L1 could be detected in ESCC cells and TILs, and elevated PD-L1 levels were significantly correlated with survival benefits (127). The results of the clinical trial KEYNOTE-180 revealed that PD-L1 expression level was associated with the therapeutic effect of pembrolizumab. Patients with EC presenting a PD-L1 CPS ≥10 presented more survival benefits than those with a CPS <10 (128). However, in other studies, survival outcomes correlated with PD-L1 expression were the opposite. In a clinical trial of SHR-1210, an anti-PD-1 antibody, Huang et al. showed that PD-L1 expression was not significantly correlated with ORR in patients with EC (129). Hynes et al. verified that in patients with EAC, survival outcomes were worse in patients whose tumors stained positive for PD-L1 than in patients with PD-L1-negative tumors who underwent neoadjuvant chemoradiation therapy (130). In addition, Ohigashi et al. observed that even in patients with ESCC, PD-L1-positive patients exhibited a poorer prognosis, and upregulation of PD-L1 was more pronounced, with worse tumor differentiation, positive lymph node metastasis, and advanced stage of ESCC (131), indicating that PD-L1 status may be a negative predictor of prognosis for patients with EC. These controversial clinical outcomes are mainly due to the heterogeneity of PD-L1 among different samples submitted, different detection methods, and the complex interaction between the anti-tumor immune response and EC cells. In addition, the treatment of patients with EC might considerably affect the outcome, indicating that a high expression of PD-L1 was likely to be a positive biomarker for patients with EC who have undergone immunotherapy but not for patients treated with other therapies. Considering the inconsistency of PD-L1 in EC, ICI therapy might be effective in certain patients with EC presenting low PD-L1 expression, while certain patients with EC presenting high PD-L1 expression might be insensitive to the same treatment. However, the prognostic value of PD-L1 in EC remains unclear. Further clinical research is necessary to confirm this relationship.

CTLA-4 is another transmembrane receptor that shares a B7 ligand with CD28. When CTLA-4 binds to B7, T cells exhibit anergy during the negative regulation of anti-tumor immunity. To date, only one study has investigated the relationship between CTLA-4 expression and the prognosis of EC. Zhang et al. demonstrated that a high density of CTLA-4 in both TILs and EC cells is associated with shortened overall survival (28). Considering that only one study demonstrated the prognostic value of CTLA-4 in EC, the study result may deviate from the true situation, and more prospective studies are needed to determine the exact correlation between them.

Other potential prognostic biomarkers, such as IDO1, IL-8, IL-10, and TGF-β, have been reported to be associated with the therapeutic response and tumor stage in EC (132–134). In the immune microenvironment of EC, anti-tumor cytokines, such as interferon-γ and tumor-killing factors, are generally believed to be insufficient. Immune suppressor factors such as TGF-β and IL-10 are upregulated. Combining immune-promoting and immune-suppressing factors may serve as a better approach for predicting the progression and therapeutic effects of EC. At present, there is a lack of studies investigating EC immune therapy prognosis, and further research is needed to determine the mechanism involved in EC progression and explore more biomarkers with prognostic value.

### Tumor-infiltrating lymphocytes

TILs have shown great prognostic value in various solid tumors, such as breast and GI cancers (135). The degree of anti-tumor immunity is largely determined by the degree of infiltration of immune cells into the tumor tissue. Upregulation of both CD8+ and CD4+ TILs in patients with EC is associated with prolonged survival and better therapeutic outcomes of neoadjuvant chemotherapy along with surgical resection (58). Considering the crucial role of TILs in the TME in the immune response, a novel concept called “Immunoscore” was proposed, which incorporates both the TNM stage and TIL degree to serve as an essential parameter for classifying cancers (136). However, the exact mechanism by which TILs are involved in the anti-tumor immune response in EC remains under investigation.

### Tumor mutation burden

TMB is commonly defined as the total number of mutations per coding area of the tumor genome. Previous studies have shown that a high mutation burden, especially non-synonymous mutations, is likely to generate neoantigens that can be recognized by T cells to activate anti-tumor immune responses (137). TMB is highly different between various cancers, ranging from 0.001/Mb to above 400/Mb. Early studies have shown that survival outcomes may be prolonged in cancer patients with high TMB who have undergone immunotherapy, indicating that TMB has the potential to act as a predictor of immunotherapy outcome (138). Hellmann et al. conducted a clinical trial using whole exome sequencing to evaluate the influence of TMB in patients with small cell lung cancer. The results showed that patients with high TMB who were treated with ICIs exhibited a higher ORR than those with low TMB (139). Additionally, the efficacy of immunotherapy in combination with ICI therapy was better than that of ICI monotherapy in patients with high TMB. This result was in accordance with the results of early studies in patients with NSCLC treated with nivolumab (140) and patients with melanoma who had received ipilimumab therapy (141), which indicated that TMB might serve as a prognostic biomarker in patients with tumors treated with ICIs. Besides, previous scholars analyzed the association between TMB and clinical outcomes in EC patients who were treated with immunotherapy. The results suggested that EC patients in the high TMB group obtained more survival benefits (25). However, in the field of EC, few studies have investigated the association between the immunotherapy and TMB, and the number of EC patients included in studies was insufficient. Thus, the reliability of TMB as a biomarker for predicting ICI effects in EC remains unclear. Further prospective clinical studies are needed to clarify this point. Despite the potential prognostic value of TMB in predicting immune response to ICIs, TMB is not without drawbacks. Because of the high heterogeneity among various biological issues even in the same solid tumor, establishing an optimal cut-off value of TMB is challenging. Additionally, the detection of TMB was also faced with strict difficulty, which had not reached a uniform standard. At present, TMB is mainly calculated on the basis of the tumor tissue. However, generally, the number of tumor cells present in one biopsy operation cannot provide an accurate measure of TMB. To overcome this hurdle, some researchers have advocated TMB detection through blood samples. Analyzing the tumor genome from a blood sample has several advantages compared to traditional biopsy, which considers only a specific tumor site. Blood samples can be used for routine diagnosis with less susceptibility to detection bias, and they can be collected using noninvasive methods. Numerous techniques, such as allele-specific PCR and cell-free DNA, can be utilized for blood-based detection (142). Although evaluating TMB from blood samples is a robust approach approved by researchers, blood samples have limited genomic content, and the results need to be verified through clinical validation (143). In general, the correlation between TMB and the response to ICIs has yielded an exciting approach for increasing the precision of immunotherapy in cancer treatment. Nevertheless, several challenges remain. Studies investigating TMB in patients with EC are insufficient to draw convincing conclusions, and the details of the immune mechanism between TMB and ICIs need to be elucidated through prospective clinical studies in the future.

### Mismatch repair deficiency

To maintain normal biological physiological activity, regulation of cell differentiation and proliferation, cells must maintain the capacity to protect their innate genome from damage by various adverse factors. When cells are exposed to exogenous and endogenous genotoxic elements, DNA errors are likely to accumulate, which might drive disorderly cell proliferation and convert normal cells into tumor cells with significant heterogeneity; this is a common phenomenon in malignancies. When DNA damage occurs, complex cellular pathways are activated, including apoptosis, cell cycle arrest, and DNA repair, which induce apoptosis and prevent cells from transforming into malignances over time. However, impairment of self-repair capacity renders normal cells sensitive to tumor-inducing factors and gradually results in malignant transformation. In recent years, mismatch repair gene deficiency has been proven to have a high incidence in various malignancies, such as ovarian and GI cancers (144). Deficiencies in mismatch repair, also called microsatellite instability-high (MSI-H) status, have been proven to be caused by mismatch repair genes such as MLH1, MSH2, MSH6, and PMS2; they facilitate the emergence of neoantigens to activate anti-tumor responses (145). In 2015, Le et al. showed that dMMR showed prognostic value in patients with cancer. They discovered that patients with dMMR tumors could possibly benefit from PD-1 blockade therapy, exhibiting prolonged PFS (146). A previous study showed that dMMR levels correlated with the depth of invasion in ESCC (147). In addition, a phase III clinical trial led by Shitara et al. applied whole exome sequencing to analyze samples from both tumor tissue and blood of patients with gastroesophageal junction adenocarcinoma (GEJ) who had been treated with pembrolizumab or paclitaxel. The study results showed that patients in the MSI-H group had a high TMB rate. Meanwhile, patients with MSI-H treated with pembrolizumab were more likely to have better survival outcomes than those who received paclitaxel therapy alone, indicating that MSI-H may serve as a positive predictive factor for the clinical efficacy of immunotherapy (148). At present, the National Comprehensive Cancer Network (NCCN) has recommended the use of pembrolizumab as a subsequent or second-line treatment in EC with dMMR (149); however, the incidence of dMMR in EC is low, only approximately 8% (147).

Unfortunately, till date, the number of studies investigating predictive biomarkers of EC is limited. Therefore, it is important to identify novel biomarkers with prognostic value for evaluating the efficacy of immunotherapy against EC, which can facilitate the selection of eligible patients with EC for immunotherapy and foster the precision of ICI therapy in the future.

## Discussion

### Future prospects

Establishment of a novel therapeutic standard for EC is anticipated in the future. Multidisciplinary combination therapy has become a hot topic. Immunotherapy combined with surgery, targeted therapy, and chemoradiotherapy has been validated in some malignancies, such as NSCLC and melanoma (150, 151). However, immunotherapy in the field of EC has a long way to go.

Common multimodal immune therapies include PD-1 inhibitors and chemotherapy. A phase III clinical trial, KEYNOTE-590, led by Kato et al., is ongoing among patients with advanced EC treated with pembrolizumab in combination with chemotherapy (152). Kraak et al. showed that GI cell lines treated with 5-fluorouracil (5-FU) chemotherapy usually have increased PD-L1 expression levels. Their results suggest an alternative mechanism of traditional immune-mediated upregulation and indicate that the combination of 5-FU with a PD-L1 inhibitor may ameliorate the clinical outcomes and improve survival benefits in patients with EC (153).

The CheckMate-032 clinical trial, led by Janjigian et al., enrolled 160 patients with metastatic EC. Patients in the study received the PD-1-blocking nivolumab combined with the CTLA-4 inhibitor ipilimumab. Patients who received combined therapy showed a better clinically meaningful anti-tumor outcome, with higher PFS rates and prolonged durable responses, compared with patients treated with nivolumab alone. However, the adverse event rate of the combination therapy was reported to be more frequent and severe than that of nivolumab monotherapy (48).

**Figure 3:**
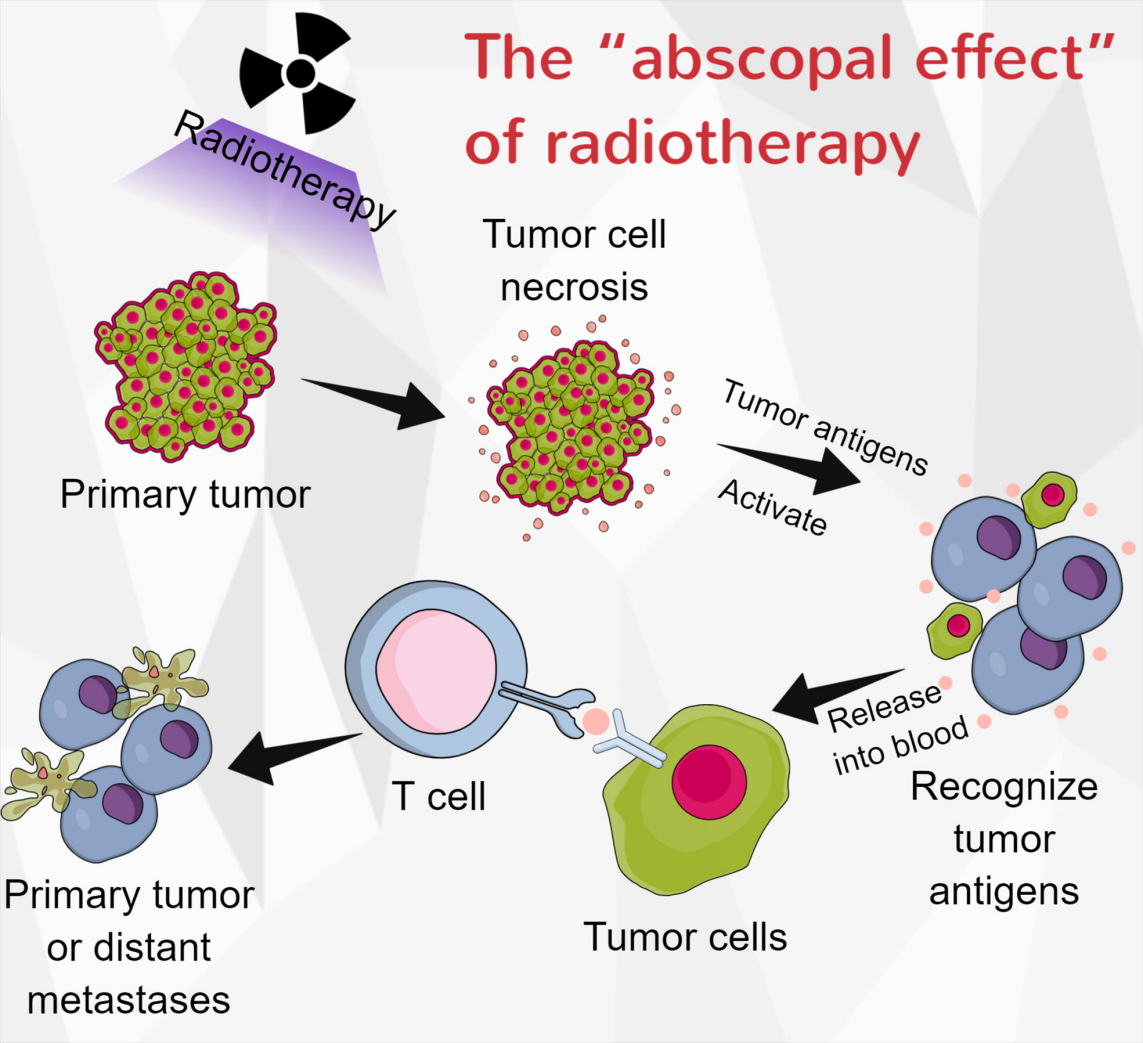
The “abscopal effect” of radiotherapy. When tumor-cell necrosis is induced by radiotherapy, the antigens with the cells are released into the blood, and they can be recognized by immune cells. These activated immune cells could then eliminate the primary tumor or distant metastases.

Radiotherapy plays a predominant role in the multidisciplinary treatment of ESCC. Radiotherapy can induce tumor cell necrosis and release antigens into the peripheral blood, which is a prerequisite for activating the anti-tumor immune response. Zhang et al. proved that immunotherapy plus radiotherapy had manageable toxicity and antitumor efficacy in patients with ESCC (154). They also demonstrated that combining concurrent chemoradiotherapy and camrelizumab had a promising antitumor effect and manageable safety in locally advanced ESCC patients (155). Interestingly, radiotherapy may partly or completely eliminate tumors outside of the radiation range, and this effect was called the “abscopal effect” (156) (**Figure 3**). In patients with melanoma, the combination of radiotherapy and CTLA-4 can induce abscopal effects (157). Park et al. conducted preclinical studies to establish EC models. They found that PD-1 inhibitors enhanced the abscopal effects of radiotherapy (157). However, radiation might also elevate PD-L1 levels in tumor cells and lead to radiotherapy resistance (158). The impact of immunotherapy in combination with radiotherapy on EC is largely uninvestigated, and it requires further investigation.

## Conclusion

In this review, we describe the current status of immunotherapy for EC and provide a clear depiction of biomarkers with prognostic value in patients with EC who have undergone immunotherapy. We additionally discuss novel strategies based on the immune environment for enhancing the current treatment effect of EC and the underlying molecular mechanisms of immunosuppression. In clinical practice, immunotherapy is commonly used as salvage therapy for patients with late-stage EC. More clinical trials are needed to verify whether immunotherapy can achieve better efficiency in early-stage applications. Because of the divergence among immune environments, it is necessary to elucidate the possible mechanisms of immunosuppression in EC so that precise targeted therapies can be developed for overcoming immunotherapy resistance in EC and for improving the prognosis of patients with EC.

## Author contributions

PF, JZ, and ZL have contributed equally. PF, conception, manuscript preparation, data collection, manuscript editing, and manuscript review. JZ, conception, manuscript editing and manuscript review. ZL, manuscript editing and manuscript review. YSY, SL, XX, XL, HZ, QS, and XZ manuscript review. YY, conception, manuscript editing and manuscript review. All authors contributed to the article and approved the submitted version.

## Funding

This work was supported by the National Nature Science Foundation of China (81970481, 82000514), the Sichuan Science and Technology Program (2022YFS0048), the 1.3.5 Project for Disciplines of Excellence, West China Hospital, Sichuan University (2020HXFH047, ZYJC18010 and 20HXJS005, 2018HXFH020), and the China Postdoctoral Science Foundation (2020M673241).

## Conflict of interest

The authors declare that the research was conducted in the absence of any commercial or financial relationships that could be construed as a potential conflict of interest.

## Publisher’s note

All claims expressed in this article are solely those of the authors and do not necessarily represent those of their affiliated organizations, or those of the publisher, the editors and the reviewers. Any product that may be evaluated in this article, or claim that may be made by its manufacturer, is not guaranteed or endorsed by the publisher.
